# Association of dietary calcium, magnesium, and vitamin D with type 2 diabetes among US adults: National health and nutrition examination survey 2007–2014—A cross‐sectional study

**DOI:** 10.1002/fsn3.2118

**Published:** 2021-01-27

**Authors:** Imran Ullah Shah, Aysha Sameen, Muhammad Faisal Manzoor, Zahoor Ahmed, Jian Gao, Umar Farooq, Sultan Mehmood Siddiqi, Rabia Siddique, Adnan Habib, Changhao Sun, Azhari Siddeeg

**Affiliations:** ^1^ Department of Nutrition and Food Hygiene College of Public Health Harbin Medical University Heilongjiang China; ^2^ Faculty of Food Nutrition and Home Sciences National Institute of Food Science and Technology University of Agriculture Faisalabad Pakistan; ^3^ School of Food and Biological Engineering Jiangsu University Zhenjiang China; ^4^ School of Food Science and Engineering South China University of Technology Guangzhou China; ^5^ University Institute of Diet and Nutritional Sciences The University of Lahore Islamabad Pakistan; ^6^ Department of Chemistry Government College University Faisalabad Faisalabad Pakistan; ^7^ Department of Human Nutrition The University of Agriculture Peshawar Peshawar Pakistan; ^8^ Department of Food Engineering Faculty of Engineering University of Gezira Wad Medani Sudan

**Keywords:** cross‐sectional study, dietary calcium, magnesium, NHANES, T2DM, vitamin D

## Abstract

Higher dietary intake of calcium (Ca), magnesium (Mg), and vitamin D has been associated with reduced risk of type 2 diabetes (T2DM), and a higher intracellular ratio of Ca to Mg leads to insulin resistance. Previous epidemiological studies did not examine the combined effects of dietary Ca, Mg, and vitamin D as well as ratio of Ca to Mg with T2DM. Therefore, we assessed the relationship between dietary intakes of Mg, Ca, and vitamin D (using 24‐hr recalls) individually and in composite and T2DM in the National Health and Nutrition Examination Survey 2007–2014, which involved 20,480 adults (9,977 men and 10,503 women) with comprehensive information on related nutrients, and anthropometric, demographic, and biomarker variables using multivariable logistic regression. The results indicated that dietary calcium at Q3 (812 mg/day) was significantly linked with T2DM in women (OR: 1.30; 95% CI: 1.02, 1.65). Dietary vitamin D at Q3 (5.25 μg/day) significantly reduced the odds of T2DM by 21% in men (OR: 0.79; 95% CI: 0.64, 0.98). This is an interesting study that has important implications for dietary recommendations. It is concluded that US adults having dietary Ca below the RDA were associated with increased risk of T2DM in all population and women, while higher ratio of Ca to Mg was associated with increased risk of T2DM in all population and increased vitamin D intake is related to decreased risk of T2DM in men. Moreover, further research is needed to make more definitive nutritional recommendations.

## Graphical abstract

This is an interesting study that has important implications for dietary recommendations. It is concluded that dietary calcium intake level of 812 mg/day (Q3) will increase the risk of developing T2DM in the general population and women. Additionally, the findings conclude that US adults having dietary calcium below the RDA were associated with increased risk of T2DM in all population and women, while higher ratio of Ca to Mg was associated with increased risk of T2DM in all population and increased vitamin D intake was associated with decreased risk of T2DM in men. Moreover, the conclusions should probably call for more studies to confirm these results in other settings.

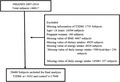

## 1 INTRODUCTION

Definition of diabetes according to the American Diabetes Association (ADA) is a group of metabolic diseases characterized by hyperglycemia resulting in either deficiency of insulin secretion, insulin action, or both (Diabetes Care, 2004). Type 2 diabetes is increasing worldwide; hence, it is a chronic disease (). The prevalence of type 2 diabetes in 2005 was 1 in 10 in every 5 adults, while that was predicted to be 1 in 3 adults by the end of 2050 (Boyle et al., 2010). T2DM is associated with poor nutrient intake (McNaughton SA, Mishra GD, Brunner EJ 2008) especially calcium and vitamin D were considered as “nutrient of concerned” (Dietary Guidelines for American (2010) while magnesium was considered as “shortfall nutrient” (United States Department of Agriculture, 2010). Dietary factors have a key role in the incidence and development of chronic diseases, especially T2DM (McNaughton et al., 2008).

Moreover, about 40% of American populations did not meet the daily requirements of calcium from their diet (US Department of Agriculture, 2015). Calcium has a vital role in the prevention of diabetes by improving insulin sensitivity and pancreatic β‐cell functions (Pittas et al., 2007). Insulin secretion depends on calcium, and thus, alterations in calcium flux adversely affect beta‐cell secretion (Pittas et al., 2007). Also, dietary calcium was inversely associated with T2DM in several epidemiological studies (Pittas et al., 2006; Villegas et al., 2009). However, some studies showed inconclusive results (De Boer et al., 2008; Kirii et al., 2009). Meta‐analysis and other research studies conducted on the American populations show a significant inverse association between risk of T2DM and low magnesium intake (Dong et al., 2011; Hruby et al., 2014). Especially, magnesium status is low in populations that eat processed diets; therefore, there is a need to explore the concerns of suboptimal magnesium status because low magnesium was related to the threat of type 2 diabetes (Rosanoff et al., 2016).

Potentially, cellular glucose metabolism is directly regulated through magnesium by affecting rate‐limiting enzymes of glycolysis (Musso, 2009). Magnesium significantly reduces the risk by 36% in Japanese men and women when compared to highest (303 mg/day) quartile versus lowest (158 mg/day) quartile (Kirii et al., 2010). Oral magnesium supplementation of 250 and 365 mg/day for the period of 3 and 6 months decreased insulin resistance in the randomized control trial (RCT; Mooren et al., 2011). A higher intracellular ratio of Ca: Mg leads to insulin resistance and hypertension in the NHANES study (Moore‐Schiltz et al., 2015). According to the hypothesis of Roasanoff (2010), low magnesium and high calcium diet may lead to cellular calcium activation from low magnesium levels, and future studies are obligatory to realize the potential effects of the ratio of Ca to Mg (Nielsen, 2010; Rosanoff, 2010). However, foods fortified with vitamin D were the main food for vitamin D intake in the United States (Holick, 2007). Human studies, and animal and human cell experiment show the protective effects of vitamin D and T2DM (Danescu et al., 2009; Kadowaki & Norman, 1984). NHANES data indicated that serum 25‐OHD was inversely found to be in dose–response patterns in non‐Hispanic whites and Mexican Americans (Scragg et al., 2004). Vitamin D stimulates the expression of insulin receptor and bindings with the beta‐cell receptor, thus affects directly the impaired beta‐cell function and insulin resistance and indirectly changeable calcium flux and extracellular calcium through β‐cell (Pittas et al., 2007).

Using NHANES (2007‐2014), however, no previous epidemiological studies have directly examined the effect of dietary calcium, magnesium, vitamin‐D and ratio of Ca:Mg and risk of T2DM, therefore, the purpose of this study was to examine the individually as well combine effect of dietary calcium, magnesium , ratio of calcium to magnesium (Ca:Mg) and vitamin D using their relevant cutoff points (RDA, Quintiles) and T2DM in US adults, the hypothesis shows that dietary calcium, magnesium, and vitamin‐D individually and in combination would decrease the risk of T2DM.

Hence, the aim to conduct this study was to examine the potential association of dietary intake for magnesium, calcium separately and in combinations consuming their ratio (Mg:Ca), and vitamin D, with type 2 diabetes among US adults, and the previous hypothesis reported that dietary calcium, magnesium, and vitamin D separately and in grouping would decrease the risk of T2DM.

## 2 MATERIALS AND METHODS

### 2.1 Study populations

This Cross sectional Study used NHANES data sets 2007–2014 data, NHANES is US national representative survey managed by center for disease control and prevention (CDC). NHANES is a consecutive survey conducted every two years, representing one cycle of the US civilian noninstitutionalized population, and NHANES data set is publically available at http://www.cdc.gov/nchs/nhanes.htm.

### 2.2 Exclusion and inclusion criteria

**FIGURE 1:**
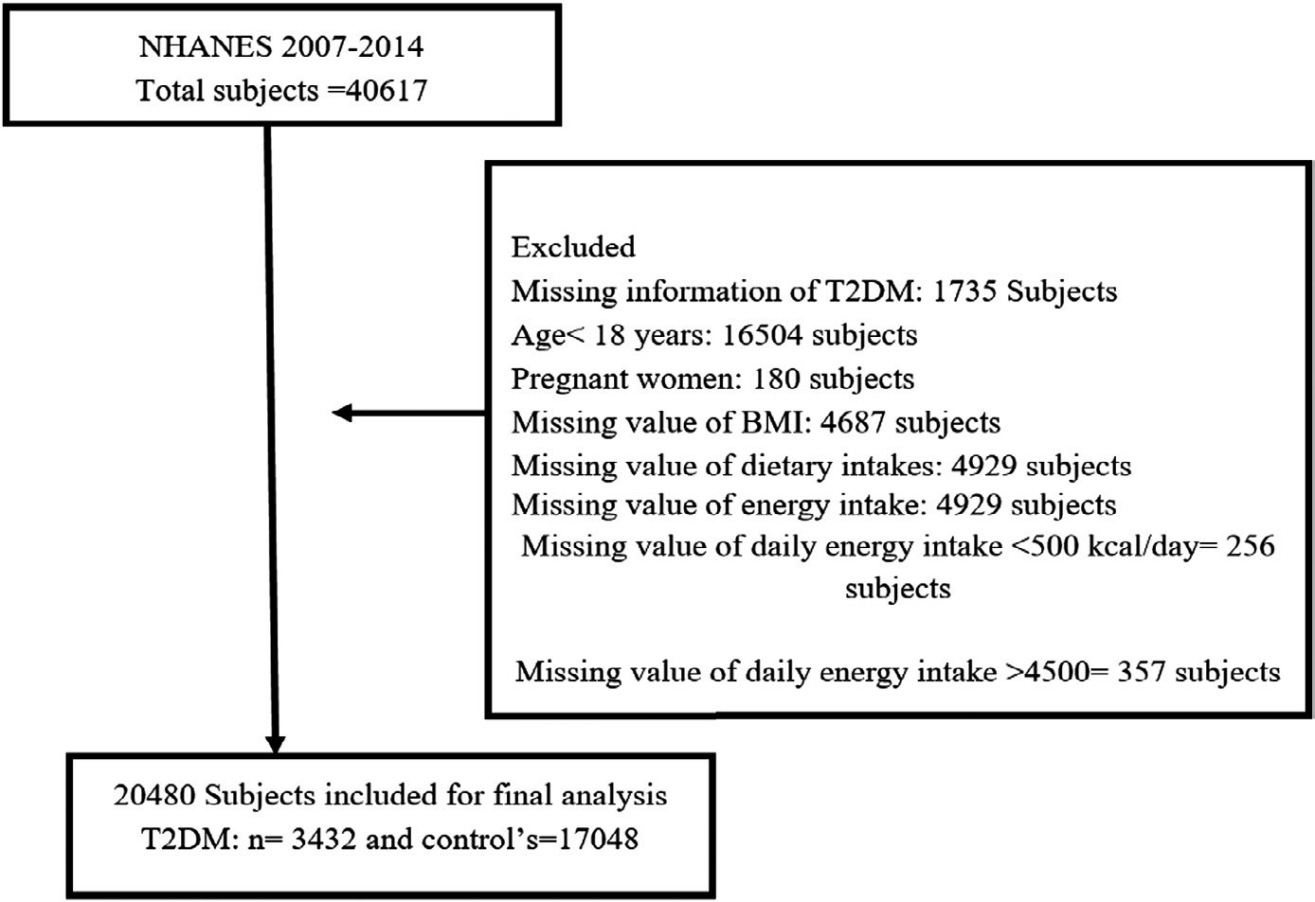
Study flowchart on selection of the participant

We included the adult's participants’ NHANES 2007–2014 data sets from mobile examination center (MEC) (*n* = 40,617) having age ≥ 18 years (men, nonpregnant women), whereas we excluded those participants having age < 18 years (16,504), pregnant women (*n* = 180), missing value daily energy intake (*n* = 4,929), daily energy intake < 500 kcal/day (*n* = 256) and > 4,500 kcal/day (*n* = 357), missing value of body mass index (*n* = 4,687), and the missing value of dietary intakes of calcium, magnesium, and vitamin D (*n* = 4,929). The detailed descriptions are also provided in Figure 1; moreover, our final data set comprised 20,480 adults (diabetes *n* = 3,432; nondiabetes *n* = 17,048).

### 2.3 Ascertainment of T2DM

The respondents diagnosed with T2DM or one of the criteria as recommended by the American Diabetes Association (Classification, 2014) will be counted as cases, having hemoglobin A1C ≥ 6.5% and/or fasting glucose ≥ 7 mmol/L and/or having diagnosed T2DM.

### 2.4 Dietary assessment

The multipass approach method was used to measure dietary intake of magnesium, vitamin D, and calcium during in‐person 24‐hr dietary recall (Moshfegh et al., 2008). This method is a precise and brief list of all foods consumed by an individual during the period of 24 hr. The second 24‐hr recall was collected by a phone call at least 3–10 days later (NHANES, 2013). Moreover, NHANES dietary interviewers' procedure manuals provided detailed descriptions about dietary interview methods (National Health and Nutrition Examination Survey, 2017).

### 2.5 Study covariates

Data about sex, age, annual household income, ethnicity, educational level, smoking, and drinking position were in use from NHANES in‐person household interviews (Zipf et al., 2013). The covariates are as follows: smoking status (used tobacco/nicotine for the last 5 days), drinking status (had at least 12 alcohol drinks/year), physical activity (yes/no), overweight, and obesity classification (Clinical Guidelines on the Identification, Evaluation, and Treatment of Overweight and Obesity in Adults, 1998). Also, total energy intakes were divided into 10 groups, while fasting glucose and glycohemoglobin (HbA1c) were used as indicators to define our outcome.

### 2.6 Statistical analysis

Data from NHANES 2007–2008, 2009–2010, 2011–2012, and 2013–2014 household interviews, dietary data, anthropometry, and laboratory data for each member were combined using unique participant identifier and took into complex sampling design. The complex and four‐stage probability sampling design was used to select participants of each NHANES cycle (NHANES, 2010). Dietary calcium, magnesium, a ratio of Ca:Mg, and vitamin D intakes were adjusted for energy intake (as a continuous variable) by the residual method explained by Willett and Stampfer) (Willett, 1998). Categorical and constant variables were presented using percentages and mean ± *SD*, respectively. Logistic regression models were used to examine the association between T2DM and calcium, magnesium, vitamin‐D and Ca:Mg intake separately and each nutrient categorized into quintiles with the lowest quintile as reference (models 1–3) and Quintile 4 as reference (model 4–6) by using logistic regression models, *p*‐value .05. Furthermore, we also stratified our models by sex and evaluated the intake of calcium and magnesium based on RDA (further down RDA or meeting the RDA based on sex‐ and age‐specific recommendations) for individual nutrient (Institute of Medicine, 2012; Institute of Medicine, 1997).

Model 1 was accustomed to sex, race, and age. Model 2 was moreover adjusted for body mass index groups (kg/m^2^), educational level, exercise regularly (yes/no), current smoker (yes/no), current drinker (yes/no), annual household income, energy intake in groups, total vitamin D (mg/day) (for the model including calcium, magnesium quintile, Ca:Mg ratio quintile), or total Ca:Mg ratio intake (mg/day) (for the model including vitamin D quintile). Model 3 was further additionally adjusted for total calcium (mg/day) (model including magnesium quintile), total calcium (mg/day) (model including magnesium quintile).

## 3 RESULTS

The study included 20,480 adults, using data from NHANES 2007–2014, which included cases (T2DM, *n* = 3,432) and noncases (nondiabetes, *n* = 17,048) (Table 1). The occurrence of type 2 diabetes in the total population was 16.8%, and women account for 48.4% of patients with type 2 diabetes. The average age was 48.4 years. The study populations with T2DM were Mexican American (9.4%), non‐Hispanic white (60.3%), non‐Hispanic black (16.4%), and other ethnic races (13.9%). The percentage of current smokers, current drinkers, exercise regularly, and family history of diabetes were 19.6%, 67.4%, 13.9%, and 3.7%, respectively. The daily mean intake of magnesium, calcium, and vitamin D, and the ratio of calcium to magnesium for individuals with diabetes were 278.9 mg/day, 890.3 mg/day, 4.7 μg/day, and 3.3 (0.04) mg/day, while daily mean intake of magnesium, calcium, vitamin D, and the ratio of calcium to magnesium for individuals with diabetes for individuals without T2DM were 300 mg, 960 mg, 4.7 μg, and 3.3 (0.02) mg/day, respectively. Only 26.9%, 14.5%, and 2.5% of individuals with T2DM met RDA of calcium, magnesium, and vitamin D.

**TABLE 1:** Selected characteristics of participants according to type 2 diabetes status in the National Health and Nutrition Examination Survey (NHANES, 2007–2014)^a^

Characteristic^b^	Diabetes (*n* = 3,432)	Nondiabetes (*n* = 17,048)	p‐value^c^
Age (year)	59.4 (0.30)	45.7 (0.30)	<0.001
Female	185 (48.4)	994 (52.6)	0.001
BMI (kg/m^2^)	33.1 (0.2)	28.2 (0.0)	<0.001
Race			
NHW	1,369 (60.3)	8,668 (68.6)	0.013
NHB	1,055 (16.4)	3,786 (10.6)	
MA	649 (9.4)	2,746 (8.3)	
Others	752 (13.9)	3,901 (12.5)	
Education			
Less than 9th grade	710 (11.4)	1834 (5.1)	<0.001
9th−11th grades	715 (15.3)	2,785 (11.3)	
High school grad./GED or equivalent	888 (24.9)	4,345 (22.2)	
Some college or AA degree	960 (30.2)	5,545 (31.0)	
College graduate or above	543 (18.0)	4,569 (30.2)	
Current smokers	652 (19.6)	4,252 (25.4)	<0.001
Current drinkers	2,161 (67.4)	12,125 (78.6)	<0.001
Exercised regularly	432 (13.9)	3,300 (20.7)	<0.001
Income			
Under $20,000	1,043 (27.3)	3,935 (14.1)	0.075
$20,000 to $45,000	1,386 (36.2)	6,098 (27.1)	
$45,000 to $75,000	627 (16.4)	3,358 (20.2)	
$75,000 to $100,000	242 (6.3)	1691 (11.5)	
Over $100,000	334 (8.7)	3,100 (23.6)	
Family history of type 2 diabetes	128 (3.7)	1714 (10.1)	<0.001
Energy intake (kcal/day)	1895.3 (21.4)	2092.28 (8.6)	<0.001
Calcium(mg/day)	890.3 (13.4)	960.36 (6.9)	<0.001
Magnesium (mg/day)	278.9 (3.5)	300.56 (2.1)	<0.001
Ratio of calcium to magnesium(mg/day)	3.3 (0.0)	3.32 (0.0)	0.335
Vitamin D (μg/day)	4.7 (0.1)	4.67 (0.0)	0.742
Above RDA calcium	923 (26.9)	5,966 (35.0)	<0.001
Above RDA magnesium	497 (14.5)	3,696 (21.7)	<0.001
Above RDA vitamin D	87 (2.5)	528 (3.1)	0.937
Fasting insulin (pmol/L)	129.2 (5.4)	71.9 (1.0)	<0.001
Fasting glucose (mmol/L)	8.65 (0.1)	5.4 (0.0)	<0.001
2‐hr glucose (mmol/L)	12.41 (0.3)	6.1 (0.0)	<0.001
HDL (mmol/L)	1.2 (0.0)	1.3 (0.0)	<0.001
TC (mmol/L)	4.8 (0.0)	5.0 (0.0)	0.015
TG (mmol/L)	1.9 (0.0)	1.3 (0.0)	<0.001
SBP (mmHg)	129.9 (0.4)	120.5 (0.2)	<0.001
DBP (mmHg)	69.0 (0.3)	70.6 (0.2)	0.001
HOMA‐IR	9.3 (0.3)	2.5 (0.0)	<0.001
HOMA‐beta	90.1 (3.5)	100.7 (7.0)	<0.001

^a^ All data analyses conducted in the present study were based on weighted estimates with sample weight provided by NHANES.

^c^ Univariate logistic regression models were used for continuous and categorical variables.

^b^ Continuous variables are presented as mean (standard error). Categorical variables are presented as *n* (%).

Table 2 shows a significant inverse association between highest quintile (median: 1502 mg) as compared to lowest quintile (median: 397 mg/day) of calcium and T2DM after adjustment of age, sex, and race (model 1:0.79; 95% CI: 0.64, 0.98, p trend: 0.042). However, this inverse association did not remain statistically significant between highest versus. lowest quintiles after further adjustment for total vitamin D, BMI groups (kg/m^2^), education level, exercise, smoking, drinking, annual household income, energy groups, and positive association observed at quintile 3 intake (Q3 812 mg/day) (model 2: OR: 1.22; 95% CI: 1.01, 1.44). A positive association was remained at quintile 3, after further adjustment for total magnesium intake (model 3: OR: 1.20; 95% CI: 1.01, 1.43). A significant inverse association remained between highest (median: 445 mg/day) versus. lowest quintiles (median: 152 mg/day) of magnesium and T2DM after alteration for race, sex, and age (model 1: OR: 0.55; 95% CI: 0.45, 0.68), and such inverse association did not remain statistically significant for model 2: OR: 1.03 95% CI (0.78, 1.36) and model 3: OR: 0.99; 95% CI (0.74, 1.33). We found a counter‐relationship among the highest quintile (445 mg/day) and magnesium versus the lowest quintile and T2DM (data not shown) when energy intake was excluded from our statistical models. The results were as follows: model 2: OR: 0.76; 95% CI: 0.60, 0.96, and model 3: OR: 0.76; 95% CI: 0.59, 0.99. We found a positive association of vitamin D at quintile 2 and T2DM when compared to Q4 reference in model 4; OR: 1.43; 95% CI: 1.14, 1.74, and such association remained at quintile 2 for model 5: OR: 1.29; 95% CI: 1.04, 1.59, and model 6: OR: 1.52; 95% CI: 1.14, 2.04. We found a significant positive association between highest (median: 4.98 mg/day) versus lowest quintiles (median: 1.76 mg/day) of calcium‐to‐magnesium ratio and T2DM after adjustment for model 1: OR: 1.23; 95% CI: 1.03, 1.48, P trend: 0.016, and such positive association remained after further adjustment for model 2: OR: 1.29; 95% CI: 1.05, 1.59, *p* trend: .010). Table 3 shows, stratification by sex revealed that association between highest (median 1457 mg/d) vs lowest (369 mg/d) quintile of dietary calcium and T2DM which shows statistically significant association only remained in female (model 1: OR: 0.71; 95% CI: 0.51, 0.99). When the model was further additionally adjusted for total vitamin‐D intake, BMI groups (kg/m^2^), education level, exercise, smoking, drinking, annual household income, Energy‐Groups which reveals the positive association at quintile 3 (812mg/d) the value of model 2: OR 1.29; 95% CI: 1.01, 1.63 that shows 1.29 times more odds of T2DM, when the model was additionally adjusted for magnesium intake such association at quintile 3 remained/ retained (model 3: OR: 1.30; 95% CI: 1.02, 1.65, *p* trend: .22).

**TABLE 2:** Prevalence of type 2 diabetes by quintiles of dietary intake in the National Health and Nutrition Examination Survey (2007–2014)

Variable	OR (95% CI) per quintile category of dietary intake^a^						*p* linear^b^
	Q1	Q2	Q3	Q4	Q5		
Calcium (mg/day)							
	397	617	812	1,051	1502		
Case/*n*	812/4,100	761/4,096	742/4,098	578/4,091	539/4,095		
Model 1	1.00	0.9 (0.7,1.1)	0.9 (0.8,1.0)	0.8 (0.6, 0.9)	0.7 (0.6, 0.9)		.042
Model 2	1.00	1.0 (0.8,1.2)	1.2 (103,1.4)	1.1 (0.8,1.4)	1.1 (0.8 ,1.5)		.169
Model 3	1.00	1.0 (0.8,1.2)	1.2 (1.0 , 1.4)	1.1 (0.8, 1.1)	1.1 (0.8, 1.5)		.226
Model 4	1.2 (1.0, 1.5)	1.1 (0.9,1.4)	1.1 (0.9,1.3)	Reference	0.9 (0.8, 1.1)		
Model 5	0.8 (0.70, 1.1)	0.9 (0.7,1.1)	1.0 (0.8,1.3)	Reference	1.0 (0.8, 1.3)		
Model 6	0.8 (0.7,1.14)	0.9 (0.7,1.1)	1.0 (0.8,1.3)	Reference	1.0 (0.8, 1.2)		
Magnesium (mg/day)							
	152	215	265	328	445		
Case/*n*	855/4,137	739/4,089	712/4,096	616/4,068	510/4,090		
Model 1	1.000	0.8 (0.7, 0.9)	0.8 (0.6, 0.9)	0.7 (0.5, 0.8)	0.5 (0.4, 0.6)		.000
Model 2	1.000	0.9 (0.7, 1.3)	1.1 (0.9, 1.3)	1.1 (0.9, 1.3)	1.0 (0.7, 1.3)		.699
Model 3	1.000	0.9 (0.7, 1.1)	1.1 (0.9, 1.3)	1.1 (0.8, 1.3)	0.9 (0.7,1.33)		.906
Model 4	1.4 (1.1, 1.7)	1.1 (1.0,1.41)	1.1 (1.0, 1.3)	Reference	0.7 (0.6 ,0.9)		
Model 5	0.8 (0.7, 1.0)	0.8 (0.71, 1.0)	1.0 (0.8 ,1.2)	Reference	0.9 (0.7, 1.1)		
Model 6	0.9 (0.7, 1.1)	0.8 (0.7, 1.0)	1.0 (0.8 ,1.2)	Reference	0.9 (0.7 ,1.1)		
	Ratio of calcium to magnesium						
	1.76	2.45	3.06	3.75	4.98		
Case/*n*	725/4,096	681/4,096	698/4,096	660/4,096	668/4,096		
Model 1	1.000	0.9 (0.8, 1.1)	0.9 (0.8, 1.1)	1.0 (0.9, 1.2)	1.2 (1.0 ,1.4)		.016
Model 2	1.000	1.0 (0.8, 1.1)	1.0 (0.8, 1.2)	1.1 (0.9, 1.3)	1.2 (1.0, 1.5)		.010
Vitamin D (μg/day)							
	1.40	3.45	5.20	7.45	12.70		
Case/*n*	1,320/7,892	848/4,639	541/3,176	430/2,808	293/1,965		
Model 1	1.000	1.0 (0.9, 1.2)	0.9 (0.7, 1.0)	0.7 (0.6 ,0.9)	0.9 (0.7 ,1.0)	.046	
Model 2	1.000	1.1 (0.9, 1.2)	0.8 (0.7, 1.0)	0.8 (0.6, 1.0)	1.0 (0.7, 1.3)	.605	
Model 3	1.000	1.1 (0.9 ,1.2)	0.8 (0.7 ,1.0)	0.8 (0.6 ,1.0)	1.0 (0.7, 1.3)	.567	
Model 4	1.1 (1.1, 1.6)	1.4 (1.1, 1.7)	1.2 (1.0, 1.4)	Reference	1.20 (0.9, 1.5)		
Model 5	1.1 (0.9, 1.4)	1.2 (1.0, 1.5)	1.0 (0.8, 1.2)	Reference	1.2 (0.9, 1.5)		
Model 6	1.1 (0.9 ,1.4)	1.2 (1.0, 1.5)	1.0 (0.8, 1.2)	Reference	1.1 (0.9, 1.5)		

^a^ Model 1 was adjusted for age, gender and race; model 2 was additionally adjusted for BMI groups (kg/m^2^), education level, exercise regularly (yes/no), current smoker (yes/no), current drinker (yes/no), annual household income, Energy‐Groups, total vitamin‐D (for the model including calcium, magnesium quintile, Ca:Mg ratio quintile) or total Ca:Mg ratio intake (for the model including Vitamin‐D quintile) Model 3 was additionally adjusted for total calcium (model including magnesium quintile), total calcium for the (model including magnesium quintile).

^b^ Model included the median value for each quintile of consumption as a continuous variable.

**TABLE 3:** Prevalence of type 2 diabetes by quintiles of dietary intake by gender in the National Health and Nutrition Examination Survey (2007–2014)

	OR (95% CI) per quintile category of dietary intake^a^					*p* linear^b^
	Q1	Q2	Q3	Q4	Q5	
Male						
Calcium (mg/day)						
mg/day	397	618	816	1,058	1538	
Case/*n*	365/1683	372/1722	347/1865	333/2146	358/2561	
Model 1	1.000	1.0 (0.8, 1.3)	0.8 (0.6, 1.1)	0.8 (0.6, 1.0)	0.8 (0.6, 1.0)	.093
Model 2	1.000	1.2 (0.9, 1.6)	1.1 (0.9, 1.5)	1.1 (0.8 ,1.6)	1.3 (0.9, 1.9)	.295
Model 3	1.000	1.2 (0.90,1.64)	1.1 (0.8, 1.5)	1.1 (0.8, 1.5)	1.2 (0.8, 1.8)	.391
Model 4	1.1 (0.9,1.5)	1.2 (0.9 ,1.7)	1.0 (0.8, 1.3)	Reference	1.0 (0.8, 1.3)	
Model 5	0.8 (0.6,1.1)	1.0 (0.7, 1.4)	0.9 (0.7, 1.2)	Reference	1.12 (0.8, 1.4)	
Model 6	0.8 (0.6,1.1)	1.0 (0.7, 1.1)	0.9 (0.7, 1.2)	Reference	1.0 (0.8, 1.4)	
Magnesium (mg/day)						
mg/day	153	216	268	329	451	
Case/*n*	338/1416	322/1615	362/1900	372/2244	381/2802	
Model 1	1.000	0.9 (0.6, 1.2)	0.8 (0.6, 1.1)	0.7 (0.5 ,0.9)	0.6 (0.4 ,0.8)	.01
Model 2	1.000	1.1 (0.8, 1.5)	1.2 (0.9, 1.7)	1.2 (0.9, 1.7)	1.2 (0.8 ,1.8)	.566
Model 3	1.000	1.1 (0.7, 1.5)	1.2 (0.9, 1.7)	1.2 (0.8 ,1.7)	1.1 (0.7 ,1.7)	.787
Model 4	1.3 (1.0,1.7)	1.2 (0.9, 1.5)	1.1 (0.9, 1.4)	Reference	0.8 (0.6, 1.0)	
Model 5	0.7 (0.5 ,1.0)	0.8 (0.6 ,1.1)	1.0 (0.8, 1.2)	Reference	09 (0.7 ,1.2)	
Model 6	0.8 (0.5,1.1)	0.8 (0.6, 1.1)	1.0 (0.8, 1.2)	Reference	0.9 (0.7 ,1.1)	
Ratio of calcium to magnesium						
mg/day	1.73	2.44	3.06	3.75	5.01	
Case/*n*	427/2243	346/1972	341/1919	324/1873	337/1970	
Model 1	1.000	1.01 (0.8 ,1.2)	0.9 (0.7, 1.1)	1.0 (0.8, 1.2)	1.2 (0.9, 1.5)	.173
Model 2	1.000	0.9 (0.7, 1.2)	0.8 (0.6, 1.1)	0.9 (0.7, 1.2)	1.0 (0.7, 1.3)	.848
Vitamin D						
μg/day	1.4	3.5	5.25	7.50	12.70	
Case/*n*	627/3504	428/2174	278/1558	240/1521	202/1220	
Model 1	1.000	1.1 (0.9 ,1.3)	0.8 (0.6, 1.0)	0.6 (0.5, 0.9)	0.9 (0.7, 1.2)	.177
Model 2	1.000	1.1 (0.9, 1.4)	0.7 (0.6, 0.9)	0.7 (0.5, 1.0)	1.00 (0.7, 1.4)	.333
Model 3	1.000	1.1 (0.9 ,1.4)	0.7 (0.6, 0.9)	0.7 (0.5, 1.0)	0.9 (0.7, 1.3)	.299
Model 4	1.4 (1.0,1.8)	1.6 (1.2, 2.0)	1.2 (0.9, 1.5)	Reference	1.4 (1.0, 1.8)	
Model 5	1.2 (0.9,1.7)	1.5 (1.1, 2.0)	1.0 (0.7, 1.3)	Reference	1.2 (0.9, 1.8)	
Model 6	1.2 (0.9,1.7)	1.5 (1.1, 2.0)	1.0 (0.7, 1.3)	Reference	1.2 (0.9, 1.7)	
Female						
Calcium (mg/day)						
mg/day	369	615	809	1,042	1,457	
Case/*n*	447/2,417	389/2,374	395/2,233	245/1,945	181/1,534	
Model 1	1.000	0.8 (0.6 ,1.0)	0.9 (0.8, 1.2)	0.7 (0.5, 1.0)	0.7 (0.5, 0.9)	.179
Model 2	1.000	0.8 (0.7, 1.1)	1.2 (1.0, 1.6)	1.1 (0.7, 1.5)	1.0 (0.7, 1.5)	.227
Model 3	1.000	0.8 (0.7, 1.1)	1.3 (1.0, 1.6)	1.1 (0.7, 1.5)	1.0 (0.7, 1.5)	.225
Model 4	1.2 (0.9,1.6)	1.0 (0.8, 1.3)	1.2 (1.0, 1.5)	Reference	0.9 (0.6 ,1.2)	
Model 5	0.9 (0.6,1.2)	0.8 (0.5, 1.0)	1.1 (0.8, 1.5)	Reference	0.9 (0.6, 1.3)	
Model 6	0.8 (0.6,1.2)	0.7 (0.5 ,1.0)	1.1 (0.8, 1.5)	Reference	0.9 (0.6, 1.3)	
Magnesium (mg/day)						
mg/day	151	214	265	326	431	
Case/*n*	517/2721	417/2474	350/2196	244/1824	129/1288	
Model 1	1.000	0.7 (0.6 ,0.9)	0.7 (0.6 ,0.9)	0.6 (0.5, 0.8)	0.4 (0.3, 0.6)	.00
Model 2	1.000	0.8 (0.6, 1.1)	1.0 (0.8, 1.3)	1.0 (0.7, 1.5)	0.9 (0.6, 1.4)	.936
Model 3	1.000	0.8 (0.6, 1.1)	1.0 (0.8, 1.3)	1.0 (0.7 ,1.5)	0.9 (0.5 ,1.4)	.972
Model 4	1.4 (1.1,1.9)	1.1 (0.8, 1.5)	1.1 (0.9, 1.4)	Reference	0.7 (0.5, 0.9)	
Model 5	0.9 (0.6,1.3)	0.8 (0.5, 1.1)	1.0 (0.7, 1.3)	Reference	0.8 (0.6, 1.2)	
Model 6	0.9 (0.6,1.3)	0.8 (0.5, 1.1)	1.0 (0.7, 1.3)	Reference	0.8 (0.6 ,1.2)	
Ratio of calcium to magnesium						
mg/day	1.79	2.47	3.05	3.74	4.96	
Case/*n*	298/1853	335/2142	335/2124	357/2177	336/2223	331/21,26
Model 1	1.000	0.9 (0.7, 1.2)	1.0 (0.8, 1.3)	1.1 (0.9, 1.4)	1.2 (0.9, 1.6)	.015
Model 2	1.000	0.9 (0.70 1.3)	0.9 (0.7, 1.3)	1.0 (0.7, 1.4)	1.0 (0.7, 1.4)	.597
Vitamin D						
μg/day	1.45	3.45	5.20	7.40	12.555	
Case/*n*	693/4388	420/2465	263/1618	190/1287	91/745	
Model 1	1.000	1.0 (0.8, 1.2)	0.9 (0.7 ,1.2)	0.8 (0.6,0.9)	0.7 (0.5, 1.1)	.096
Model 2	1.000	1.0 (0.8, 1.2)	1.0 (0.7, 1.3)	1.0 (0.7, 1.2)	1.0 (0.6, 1.7)	.812
Model 3	1.000	1.0 (0.8, 1.2)	1.0 (0.7 ,1.3)	1.0 (0.7, 1.2)	1.0 (0.6, 1.7)	.244
Model 4	1.2 (1.0,1.4)	1.2 (0.9, 1.5)	1.1 (0.9, 1.5)	Reference	0.9 (0.6, 1.4)	
Model 5	0.9 (0.7,1.2)	1.0 (0.8, 1.3)	1.0 (0.7, 1.4)	Reference	1.0 (0.6 ,1.6)	
Model 6	0.9 (0.7,1.2)	1.0 (0.8, 1.2)	1.0 (0.7, 1.4)	Reference	1.0 (0.6, 1.6)	

^a^ Model 1 was adjusted for age, race, BMI groups (kg/m^2^), education level, exercise regularly (yes/no), current smoker (yes/no), current drinker (yes/no), and annual household income; model 2 was adjusted for total vitamin D (for the model containing calcium, magnesium quintile, Ca:Mg ratio quintile). Model 3 was adjusted for total calcium (model together with magnesium quintile), total calcium for the (model including magnesium quintile)

^b^ Model included the median value for each quintile consumption as a continuous variable.

A significant inverse association was found for women across dietary magnesium quintile and T2DM (model 1: OR: 0.47; 95% CI: 0.33, 0.67, and *p* trend: .00). Results showed a significant inverse association in women and men between highest (median: 431 mg/day) versus. lowest quintiles (median: 151 mg/day) of magnesium and T2DM after adjustment of age, sex, and race (model 1: OR: 0.47; 95% CI: 0.33, 0.67 *p* trend: .00), while in men (model 1: OR: 0.63; 95% CI: 0.47, 0.85 *p* trend: .01), respectively, such inverse association did not exist further in models 2 and 3. Dietary vitamin D in male has significantly reduced the odds of T2DM at quintile 4 (7.50 μg/day) (model 1: OR: 0.69; 95% CI: 0.53, 0.91), and we found that vitamin D intake at quintile 4 (7.50 μg/day) reduced the odds of T2DM in men (model 1: OR: 0.69; 95% CI: 0.53, 0.91), after further adjustment at quintile 3 (5.25 μg/d) (model 2: OR: 0.79; 95% CI: 0.64, 0.98), which shows inverse association to reduce the odds of T2DM. Furthermore, vitamin D intake at quintile (Q2) as compared to Q4 (reference) contains the recommended amount for model 4: OR: 1.61; 95% CI: 1.24, 2.09, model 5: OR: 1.53; 95% CI: 1.14, 2.04, and model 6: OR: 1.52; 95% CI: 1.14, 2.04, which revealed that T2DM participants at quintile 2 (3.5 μg/day) had more odds of T2DM when compared with reference Q4 (7.50 μg/day); however, no association was found for Ca: Mg ratio in men and women.

Several arrangements of intake for vitamin D and Ca and Mg used RDA (cutoff points, Table 4). Results showed no significant association was found in all populations and those who met the RDA for calcium depend on age (1,000–1,200 mg/day) (OR: 0.97; 95% CI: 0.76, 1.24), magnesium (310–320) (OR: 1.17; 95% CI: 0.97, 1.42), and vitamin D ≥ 15 μg/(600 IU/day) (OR: 1.10; 95% CI: 0.63, 1.92). By sex‐specific analysis for the evaluation of OR 95% CI as well multiplicative interactions between sex and those subjects meeting the RDA for Ca and Mg and vitamin‐D (shown in Table 4) that reveals non‐significant interaction between sex and meeting the RDA for Ca, Mg and vitamin‐D.

**TABLE 4:** OR for being below the RDA or meeting or being above the RDA for calcium and magnesium combinations for all individuals (odds ratios and 95% confidence intervals)

	All			Female			Male		
	OR^a^	95% CI	*p*	OR^a^	95% CI	*p*	OR^a^	95% CI	P
Below Mg RDA^b^	1.00	Ref.		1.00	Ref.		1.00	Ref.	
Met Mg RDA	0.97	0.7–1.2	.83	0.88	0.2–2.7	.82	0.97	0.7–1.2	0.92
P_interaction term_ (sex × Mg_RDA)^e^			<.001						
Below Ca RDA^c^	1.00	Ref.		1.00	Ref.		1.00	Ref.	
Met Ca RDA	1.17	0.9–1.4	.08	1.08	0.8–1.4	.55	1.25	0.9–1.6	0.07
P_interaction term_ (sex × Ca_RDA)			.53						
Below VD RDA^d^	1.00	Ref.		1.00	Ref.		1.00	Ref.	
Met VD RDA	1.10	0.6–1.9	.71	1.25	0.4–3.8	.68	1.08	0.6–1.9	0.78
P_interaction term_ (sex × VD_RDA)			.84						
Met Ca RDA and met Mg RDA	1.18	0.9–1.4	1.11	0.88	0.5–1.4	.61	1.28	0.9–1.6	0.051
P_interaction term_ (sex × Mg _Ca _RDA)			.89						

Note

Ref., reference values.

^a^ Model was adjusted for gender, age, race, BMI groups (kg/m^2^), education level, exercise regularly (yes/no), current smoker (yes/no), current drinker (yes/no), annual household income, BMI groups (kg/m^2^) total energy intake groups (kcal/day), total vitamin D (for the model including RDA of calcium and magnesium), total calcium (for the model including RDA of vitamin D and magnesium), and adjusted for total magnesium (for the model including RDA of vitamin D and calcium).

^b^ RDA for Mg is 400 mg/day for males aged 18–30 years and 420 mg/day for males > 30 years of age; RDA for Mg is 310 mg/day for females aged 18–30 years and 320 mg/day for females > 50 years of age.

^c^ RDA for calcium is 1,000 mg/day for males aged 20–70 years and 1,200 mg/day for males > 70 years of age; RDA for Ca is 1,000 mg/day for females aged 20–50 years and 1,200 mg/day for females > 50 years of age.

^d^ RDA for vitamin D is 15 μg/day (600 IU) for both males and females aged ≤ 70 years and RDA > 15 μg/day (>600 IU/day) for aged ≥ 71 for both males and females.

^e^ *p*‐value for the multiplicative interaction term.

## 4 DISCUSSION

To our information, this is the first study aimed to assess the combined effect of dietary calcium, magnesium, Ca: Mg intake ratio, and vitamin D on T2DM using 8 years of NHANES data (2007–2014). It is concluded that dietary calcium intake of 812 mg/day (below RDA) increased the odds by 1.20 times, while the calcium‐to‐magnesium ratio between highest versus lowest quintiles increased the odds by 1.29 times in all populations. Calcium intake (809 mg/day, below RDA) increased the odds by 1.30 times in women, while vitamin D intake (5.25 μg/day) reduced the odds by 21% in men. Moreover, the importance of RDA indicated vitamin D intake when compared with the reference (Q4 recommended intake), and the odds of T2DM increased by 1.29 times in Q2 in both genders while 1.52 times in men.

There was no relationship between the lowest versus highest quintiles of dietary intake of calcium, magnesium, and vitamin D and T2DM. Mostly, studies reported that one‐third of Americans did not meet adequate intake (AI) for calcium (Bailey et al., 2010; USDA, 2005), while some studies showed that about half of the US populations did not meet the RDA of magnesium (Moshfegh et al., 2008; Nicklas et al., October, 2014; USDA, 2005). Similar findings reported low dietary intake of magnesium, calcium, and vitamin D below RDA level. Moreover, the results for dietary calcium intake in our conclusions are stable with the results of dietary calcium intake between highest (629 mg/day) versus lowest quartiles (254 mg/day) with multivariable odds ratio of 0.93; 95% CI: 0.71–1.22, for men and women, 810 versus. 365, 0.76; 95% CI: 0.56–1.03 (Kirii et al., 2009). Similar study were conducted in Australia the relative risk RR; 0.94; 95%; CI: 0.61 ‐1.46 for highest (1,060–2,317) versus. lowest (171–740) (Gagnon et al., 2011). However, no association between calcium and diabetes were found and such association disappeared (RR: 1.04, 95% CI: 0.88–1.24). However, inverse association with magnesium remained, RR: 0.65, 95% CI: 0.54–0.78 (Van Dam et al., 2006), and similarly, studies reported the same results (Gagnon et al., 2011; Pittas et al., 2006; Van Dam et al., 2006).

The culprit mechanism in our study for such observed association) as vitamin‐D facilities intestinal calcium absorption and insufficient calcium (due to vitamin‐D insufficiency, low calcium intake) because calcium is required in regulating glucose intolerance due to vitamin‐D deficiency in vivo (Beaulieu et al., 1993).

The results of the meta‐analysis show 600 mg/day calcium intake as desirable, while 1,200 mg daily intake was considered to be preferred (Pittas et al., 2006). Some studies showed a nonsignificant association between dietary magnesium and diabetes (Hopping et al., 2010; Wang et al., 2005). Likewise, the intake of magnesium in our study was low as the intake in the national sample of African American women (median: 183 mg/day) that is below the RDA (320 mg/day) (Ford & Mokdad, 2003). Also, in Mg and glucose metabolism the results are inconsistent (De Valk, 1999). Our study have similar finding with study conducted for 5 years which showed dietary magnesium highest (348 mg/day) versus. lowest quintile (213mg/day) men, RR; 0.86; 95 % CI; 0.63‐1.16 while the results for women RR; 0.92; 95% CI: 0.66–1.28 (Nanri et al., 2010). The ARIC study conducted in the United States for 6 years found a nonsignificant association between dietary magnesium highest (418 mg/day) versus. lowest quintiles (308 mg/day) in the black subjects, RR: 1.05; (0.58, 1.93) but not in white (Kao et al., 1999). Our results had a significant association among race (*p* trend = .013), while one of the studies reported ethnic differences to exist among NHANES participants (Scragg et al., 2004). Also, a nonsignificant association between dietary magnesium and diabetes, OR: 0.73; 95% CI: 0.51, 1.04, conclusions from the Melbourne collaborative cohort study (Hodge et al., 2004; Li et al., 2009), supports our findings. The results of our study are generally consistent with previous cross‐sectional studies conducted on non‐Hispanic whites and Mexican Americans aged ≥ 20 years and found an inverse relationship between vitamin D and diabetes (Scragg et al., 2004). Our study reported dietary vitamin D at quintile 3 reduced the odds of T2DM by 21% only among men. Nurses’ health study shows vitamin D intake > 800 IU and calcium > 1,200 reduced T2DM risk by 33% as compared to calcium intake < 600 mg/day and vitamin D < 400 IU/day, RR: 0.67; 95% CI: 0.49–0.90 (Pittas et al., 2006). The result of our study matched with finding RR; 0.87;95% CI; 0.75‐1.00 when the model were adjusted for several factors including dietary magnesium, retinol, and calcium, a non‐significant association was observed between vitamin‐D3 intake and T2DM (Pittas et al., 2006), while similar findings from another nested case–control study (Robinson et al., 2011). The findings of our study showed that 60% of participants were non‐Hispanic white and few participants fall in our highest quintile (Q5). Conversely, overweight is still a well‐known risk factor for T2DM in many studies (Colditz et al., 1995). The results of other studies indicated that genetic variants of dietary magnesium channels such as TRPM6 and TRPM7 increased the incidence risk of T2DM when the dietary intake for magnesium is less than 250 mg/day (Song et al., 2009). Higher calcium‐to‐magnesium ratio leads to inappropriate cellular calcium activation, which leads to T2DM, CVD, and other diseases if the magnesium deficiency is not corrected (Rosanoff, 2010). In this study, first time we evaluated the individual and combined effects of magnesium, calcium, vitamin D, and Ca: Mg ratio using 8 years of NHANES data, which are the US nationally large representative sample, which demonstrates the strength of this study; secondly, adjustment for several confounding factors; thirdly, the trained staff with standardized protocols of NHANES and efficacy and precision of the data; and fourthly, the criteria been used for case ascertainment ensured all cases were included in the study. However, processed foods, soda/ soft drinks, beverages, poor nutrient choices, and unhealthy diet will not able the American to meet the daily recommended intake of these micronutrients and health professionals need strategies and dietary interventions, which are an important aspect of epidemiology and public health (O’Neil et al., 2012).

## 5 LIMITATIONS

The cross‐sectional study does not allow the formation of a temporal relationship. United States have 14% gestational diabetes ratio due to that we excluded gestational diabetes cases from our study (BA, I, JC, & WPT, 2004). Moreover, dietary supplement data inevitably have missing values which is why we do not include supplementation data. However, intake was done by the recall. Also, the results for the study may not be universal because the study was done in one geographic area; thus, further research is needed to make more definitive nutritional recommendations. Therefore, further prospective studies are warranted due to public health importance. Thus, essential micronutrients (vitamin D, calcium, magnesium) related to chronic ailments of global concern include T2D as their public health importance.

## 6 CONCLUSIONS

This is an interesting study that has important implications for dietary recommendations. It is concluded that dietary calcium intake level of 812 mg/day (Q3) will raise the threat of developing T2DM in the general population and women. Additionally, the findings conclude that US adults having dietary calcium below the RDA were related to an enhanced risk of T2DM in all population, while higher ratio of Ca to Mg was associated with increased risk of T2DM in all population and increased vitamin D intake was associated with decreased risk of T2DM in men. Therefore, the conclusions should probably call for further studies to confirm these results in other settings.

## CONFLICT OF INTEREST

The authors declare no conflict of interest. “The funders had no role in the design of the study; in the collection, analyses, or interpretation of data; in the writing of the manuscript; or in the decision to publish the results.”
